# Activation of PmrA inhibits LpxT-dependent phosphorylation of lipid A promoting resistance to antimicrobial peptides

**DOI:** 10.1111/j.1365-2958.2010.07150.x

**Published:** 2010-04-09

**Authors:** Carmen M Herrera, Jessica V Hankins, M Stephen Trent

**Affiliations:** 1 Section of Molecular Genetics and Microbiology, The University of Texas at AustinAustin, TX 78712, USA; 3 The Institute of Cellular and Molecular Biology, The University of Texas at AustinAustin, TX 78712, USA; 2 Department of Biochemistry and Molecular Biology, Medical College of GeorgiaAugusta, Georgia, 30912, USA

## Abstract

During its transport to the bacterial surface, the phosphate groups of the lipid A anchor of *Escherichia coli* and *Salmonella* lipopolysaccharide are modified by membrane enzymes including ArnT, EptA and LpxT. ArnT and EptA catalyse the periplasmic addition of the positively charged substituents 4-amino-4-deoxy-L-arabinose and phosphoethanolamine respectively. These modifications are controlled by the PmrA transcriptional regulator and confer resistance to cationic antimicrobial peptides, including polymyxin. LpxT, however, catalyses the phosphorylation of lipid A at the 1-position forming 1-diphosphate lipid A increasing the negative charge of the bacterial surface. Here, we report that PmrA is involved in the regulation of LpxT. Interestingly, this regulation does not occur at the level of transcription, but rather following the assembly of LpxT into the inner membrane. PmrA-dependent inhibition of LpxT is required for phosphoethanolamine decoration of lipid A, which is shown here to be critical for *E. coli* to resist the bactericidal activity of polymyxin. Furthermore, although *Salmonella* lipid A is more prevalently modified with l-4-aminoarabinose, we demonstrate that loss of *Salmonella lpxT* greatly increases EptA modification. The current work is an example of the complexities associated with the structural remodelling of Gram-negative lipopolysaccharides promoting bacterial survival.

## Introduction

Lipopolysaccharide (LPS) is a complex bacterial surface structure that is composed of lipid A, a core oligosaccharide, and the O-antigen polysaccharide. LPS is characterized by its endotoxicity, which is provided by the lipid A domain, a hydrophobic molecule that anchors LPS to the outer membrane (Raetz, 1990; Raetz and Whitfield, 2002). Given that both the lipid A domain and the inner core region of LPS can be negatively charged, ionic interactions with divalent cations such as magnesium provide stability and the relative impermeability of the Gram-negative outer membrane to hydrophobic toxic compounds (Raetz and Whitfield, 2002).

**Fig. 1:**
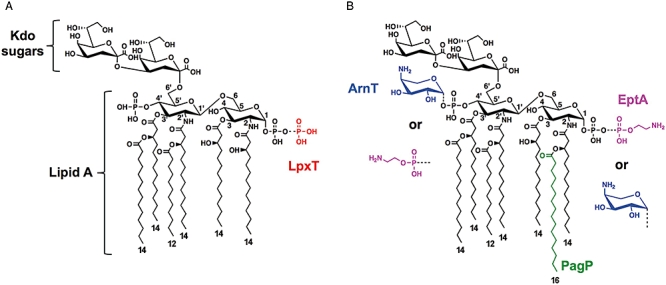
Comparison of *E. coli* Kdo_2_-Lipid A and its modified forms. Lipid A is a glucosamine-based lipid A that serves as the hydrophobic anchor of LPS. The lengths of the acyl chains are indicated and dashed bonds indicate partial substitutions. A. In *E. coli* K-12, the predominant species is hexa-acylated and phosphorylated at the 1 and 4′ positions. Two Kdo residues from the inner core region of LPS are shown and are linked at the 6′-position of the molecule. LpxT, a phosphotransferase, adds a second phosphate group (red) to the 1-position, resulting in a 1-diphosphate (1-PP) species. B. The membrane bound enzymes ArnT and EptA can modify the lipid A phosphate groups with L-4-aminoarabinose (L-Ara4N) and phosphoethanolamine (pEtN) shown in blue and magenta respectively. Expression of ArnT and EptA (PmrC) is under the control of PmrA–PmrB. PagP, an outer membrane enzyme under the control PhoP–PhoQ, catalyses the addition of a palmitate (green) to the 2-position yielding a hepta-acylated lipid A.

The lipid A of *Salmonella* and *Escherichia coli* consists of a β-1′-6-linked glucosamine disaccharide backbone bearing six fatty acyl chains, that is phosphorylated at the 1 and 4′ positions. This molecule is further glycosylated at the 6′-position with two 3-deoxy-d-*manno*-octusolonic acid (Kdo) moieties that link the lipid A domain to the remaining polysaccharide of LPS (Fig. 1A) (Raetz, 1990; Trent, 2004). Gram-negative bacteria have evolved mechanisms to modify their basic lipid A structure in response to their environment by addition or removal of fatty acyl chains along with the alteration of phosphate groups (Trent, 2004; Raetz *et al*., 2007) (Fig. 1B). For instance, modification of the phosphate groups of *E. coli* and *Salmonella* lipid A are carried out by the enzymes ArnT and EptA, which catalyse the addition of 4-amino-4-deoxy-l-arabinose (L-Ara4N) and phosphoethanolamine (pEtN) respectively (Zhou *et al*., 1999; 2001; Trent *et al*., 2001a; Lee *et al*., 2004). In *Salmonella*, L-Ara4N is preferentially added to the 4′-phosphate group whereas pEtN is added to the 1-phosphate group of lipid A (Fig. 1B); however, these target positions can be either exchanged or modified with the same moiety if either ArnT or EptA is absent (Zhou *et al*., 2001). In *E. coli* K-12, L-Ara4N and pEtN are incorporated to lipid A only when cells are grown under specific conditions (e.g. low pH) (Zhou *et al*., 1999; Trent *et al*., 2001b; Gibbons *et al*., 2005).

Modification of lipid A with an additional phosphate group at the 1-position forming a 1-diphosphate (1-PP) species is mediated by the undecaprenyl phosphotransferase, LpxT (Touze *et al*., 2008). This enzyme exhibits two catalytic activities, a phosphatase activity for dephosphorylation of the distal phosphate group from undecaprenyl pyrophosphate (phosphate donor), and a phosphotransferase activity for transferring this phosphate to the 1-position of lipid A (El Ghachi *et al*., 2005; Tatar *et al*., 2007; Touze *et al*., 2008). The 1-diphosphate lipid A species was initially described in *Salmonella* and *E. coli*, but it has also been suggested that this is widely extended in Gram-negative bacteria (Jones *et al*., 2008; John *et al*., 2009). The absence of 1-PP species in previous studies might arise from the fact that the pyrophosphate groups have differing stabilities during mass spectrometry depending upon the type of ionization, excitation mode or detector used during analysis of lipid A analytes.

EptA, ArnT and LpxT are all inner membrane proteins, which modify lipid A following its transport to the periplasmic face of the inner membrane (Trent *et al*., 2001a; Lee *et al*., 2004; Tatar *et al*., 2007; Touze *et al*., 2008). Synthesis of EptA and ArnT is regulated by the PmrA–PmrB two-component system, where PmrA is the transcriptional regulator and PmrB its cognate sensor. PmrB is an inner membrane protein capable of sensing different signals through its periplasmic domain including iron (Fe^3+^) (Wosten *et al*., 2000; Chamnongpol *et al*., 2002; Hagiwara *et al*., 2004), zinc (Lee *et al*., 2005) and mild acid pH (pH 5.8) (Soncini and Groisman, 1996; Perez and Groisman, 2007). Other signals such as aluminium (Al^3+^) (Nishino *et al*., 2006) and vanadate (Zhou *et al*., 1999) activate PmrA–PmrB, but their exact mechanisms of induction are unknown. Lastly, PmrA is activated under Mg^2+^ limiting growth conditions or upon exposure to cationic antimicrobial peptides (CAMPs). Under these conditions PmrA activation is mediated by a second two-component system, PhoP–PhoQ. According to the proposed mechanism, activation of PhoP in *Salmonella* induces the synthesis of PmrD, which regulates PmrA activity post-transcriptionally by preventing dephosphorylation of PmrA (Kox *et al*., 2000; Kato and Groisman, 2004). It was suggested that PmrD does not exert a similar role in *E. coli* (Winfield and Groisman, 2004), but microarray analysis of Fe^3+^-induced PmrA–PmrB genes revealed that *E. coli* PmrD plays a role in a cross-regulation of genes involved in LPS modification (Hagiwara *et al*., 2004).

Activation of PmrA-dependent *arnT* and *eptA* genes promotes lipid A modification masking negative phosphate groups with positively charged moieties, with a concomitant resistance to antimicrobial peptides, and an increase in virulence (Gunn *et al*., 2000; Wosten *et al*., 2000; Tamayo *et al*., 2005a; Perez and Groisman, 2007). Lipid A isolated from *E. coli* K-12 grown in nutrient rich medium contains two-thirds of 1,4′-*bis*-phosphorylated and one-third of 1-PP lipid A species (Touze *et al*., 2008) (Fig. 1A). Since 1-PP lipid A modification might be widely distributed among Gram-negative bacteria (Jones *et al*., 2008), the proportion between these lipid A species raises questions about its regulation and biological relevance. Therefore, we focused our interest on studying regulation of *E. coli* LpxT-dependent lipid A modification. Here we report that induction of PmrA inhibits the synthesis of 1-PP modified lipid A within the bacterial membrane. Our data demonstrate that LpxT is post-translationally regulated at the membrane, thereby promoting EptA activity. Furthermore, this work highlights the critical role that pEtN modification plays in resistance to CAMPs and the complex interactions of periplasmic lipid A modification systems.

## Results

### Activation of the PmrA–PmrB two-component system inhibits LpxT-dependent lipid A modification

**Fig. 2:**
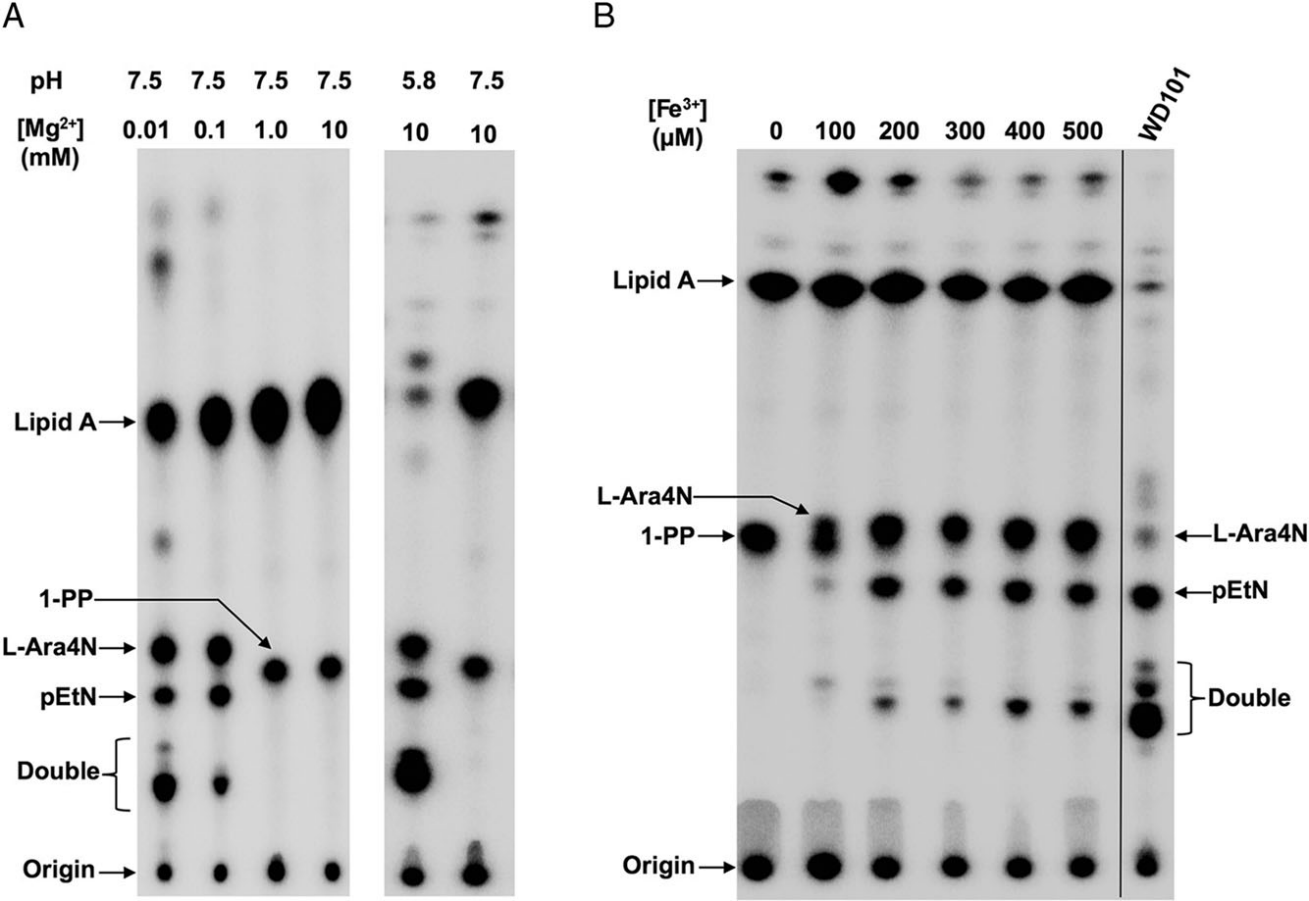
Induction of ArnT and EptA expression blocks LpxT-dependent lipid A modification. ^32^P-labelled lipid A species isolated from strain W3110 under different growth conditions were separated by TLC and visualized by phosphorimaging analysis. A. Cells were grown in N-minimal medium containing different MgCl_2_ concentrations at either pH 7.5 or 5.8. B. Cells were grown in LB medium containing increasing concentrations of FeSO_4_. Major ^32^P-lipid A species are indicated with arrows: lipid A (1,4′-*bis*-phosphorylated), 1-PP (1-diphosphate), L-Ara4N (L-4-aminoarabinose), pEtN (phosphoethanolamine) and double (modifications at 1- and 4′-positions with similar or combined substituents). The identity of lipid A species is based upon data presented herein and previous publications ((Zhou *et al*., 1999; Zhou *et al*., 2000; Zhou *et al*., 2001; Trent *et al*., 2001b). Strain WD101 (*pmrA*^C^ mutant) is used as control for PmrA-dependent lipid A modification profile. As indicated by the TLC analysis of each sample (10 000 cpm spotted·per lane), increased modification of lipid A results in decreasing levels of the 1,4′-*bis*-phosphorylated species.

PmrA–PmrB and PhoP–PhoQ two-component systems are involved in regulation of lipid A modifications in *Salmonella* and *E. coli* (Raetz *et al*., 2007; Gunn, 2008). However, regulation of LpxT, an enzyme that phosphorylates lipid A at the 1-position has yet to be elucidated. To investigate whether PmrA–PmrB and PhoP–PhoQ are involved in *lpxT* regulation, we evaluated the ^32^P-labelled lipid A profile of *E. coli* under growth conditions known to stimulate these regulatory systems. Wild-type strain W3110 was grown in N-minimal medium, pH 7.5 and supplemented with Mg^2+^ at different concentrations. ^32^P-labelled lipid A species were isolated and analysed by thin-layer chromatography (TLC) as previously described (Tran *et al*., 2004). Bacteria grown at pH 7.5 with low concentrations of Mg^2+^ (0.01–0.1 mM) produced lipid A species modified with L-Ara4N and pEtN, but lacking 1-PP (Fig. 2A). LpxT-dependent lipid A modification was restored at high Mg^2+^ (1–10 mM) concentrations (Fig. 2A). Likewise, shifting the bacteria to mildly acidic growth conditions (pH 5.8) resulted in the decrease of LpxT activity and strong induction of L-Ara4N and pEtN addition even in the presence of 10 mM Mg^2+^ (Fig. 2A). Therefore, like *Salmonella* (Gibbons *et al*., 2005), growth of *E. coli* under mildly acidic conditions results in modification of lipid A phosphate groups with amine-containing residues independent of Mg^2+^ concentration.

In *Salmonella* PhoP–PhoQ is activated by both low Mg^2+^ (Guo *et al*., 1997) and mildly acidic pH (Prost *et al*., 2007), leading to activation of PmrA (Kox *et al*., 2000; Kato and Groisman, 2004). Although the protein connector PmrD is reported to be non-functional in *E. coli* (Winfield and Groisman, 2004), there is evidence that the two systems are linked (Hagiwara *et al*., 2004). We wanted to avoid a possible cross-regulation between these two-component systems for lipid A modification under the evaluated conditions. Therefore, wild-type *E. coli* was grown in Luria–Bertani (LB) medium supplemented with increasing concentrations of FeSO_4_. Fe^2+^ is quickly oxidized to Fe^3+^ serving as a specific signal for activation of the PmrA–PmrB system (Wosten *et al*., 2000). In the absence of iron, 1,4′-*bis*-phosphorylated and 1-PP lipid A are synthesized, whereas bacteria grown in the presence of 100 µM Fe synthesized lipid A substituted with low levels of L-Ara4N, pEtN and 1-PP (Fig. 2B). Increasing iron concentrations further (200–500 µM) promoted synthesis of L-Ara4N and pEtN-lipid A species, with a loss of LpxT-dependent phosphorylation. The nature of these substitutions was confirmed by comparing the lipid A profile of the previously characterized *E. coli* strain (WD101) harbouring a PmrA constitutive (PmrA^c^) phenotype (Trent *et al*., 2001b). However, in WD101 the proportions of single and double lipid A modifications were different compared with the wild-type strain (Fig. 2B). In LB medium, 200 µM Fe^3+^ was considered as the iron concentration required for a complete activation of PmrA-dependent genes involved in lipid A modification. On the whole, any growth condition promoting activation of PmrA resulted in a near total loss of 1-PP lipid A.

### LpxT-dependent lipid A modification is not restored in *ΔarnT* or *ΔeptA* mutants

**Fig. 3:**
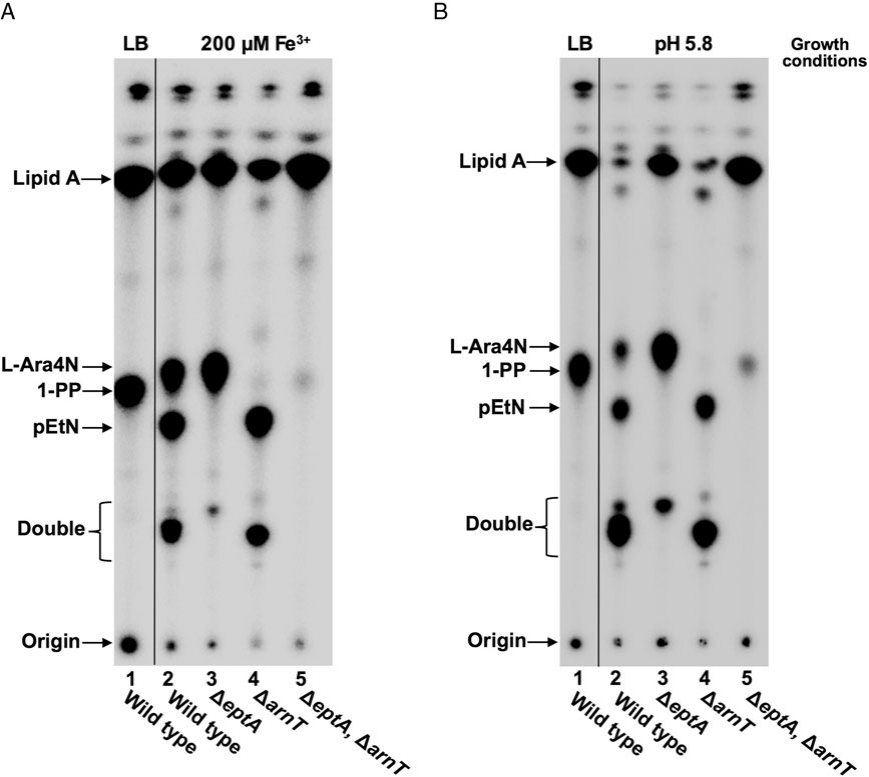
Activation of *E. coli* PmrA inhibits 1-diphosphate lipid A formation. Bacteria were ^32^P-labelled in the presence of stimuli known to induce PmrA. The indicated strains were grown in (A) LB medium containing 200 µM FeSO_4_ or in (B) N-minimal medium supplemented with 10 mM MgCl_2_, at pH 5.8. Wild-type W3110 grown in LB medium is used as control. ^32^P-lipid A species were isolated, separated by TLC, and visualized by phosphorimaging. Major lipid A species are indicated as in Fig. 2.

**Fig. 4:**
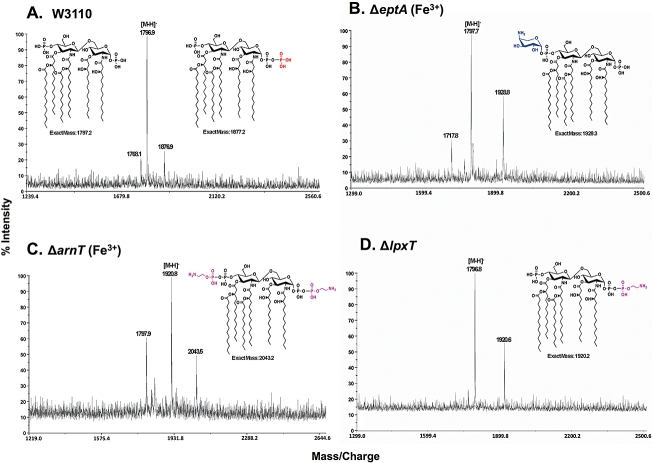
Mass spectrometry analysis of *E. coli* lipid A isolated from Δ*eptA*, Δ*arnT* and Δ*lpxT* mutants. Lipid A was isolated from the indicated strains grown in LB with or without 200 µM FeSO_4_ and analysed by MALDI-TOF mass spectrometry in the negative-ion mode. W3110 produced both *bis*-phosphorylated (m/z 1796.9) and 1-diphosphate (m/z 1876.9) lipid A species (A). During growth in Fe^3+^, Δ*eptA* and Δ*arnT* mutants synthesized L-Ara4N (m/z 1928.8, B) and pEtN (m/z 1920.8, 2043.5; C) modified forms of lipid A respectively. Even in the absence of Fe^3+^, W3110 lacking functional *lpxT* produced pEtN modified lipid A (m/z 1920.6, D). The peak at m/z 1717.8 amu in (B) arises from the removal of a phosphate group at the 1-position, which commonly occurs during mass spectrometric analysis of lipid A molecules (Tran *et al*., 2004).

We examined whether inactivation of ArnT or EptA, or both, would restore LpxT-dependent phosphorylation of lipid A. For this purpose, we created the isogenic strains CH030 (Δ*eptA*), CH040 (Δ*arnT*) and the double mutant CH034 (Δ*arnT*Δ*eptA*) in the W3110 background. ^32^P-lipid A profiles were evaluated in cells grown in LB medium supplemented with 200 µM FeSO_4_ (Fig. 3A). In CH030 (Δ*eptA*), strong induction of single L-Ara4N modification was observed (Fig. 3A, lane 3), whereas double L-Ara4N-lipid A species were only barely visible. In CH040 (Δ*arnT*), both single and double pEtN-lipid A substitutions were evident (Fig. 3, lane 4). These modifications were confirmed by matrix-assisted laser desorption/ionization-time of flight (MALDI-TOF) mass spectrometry (Fig. 4). Lipid A isolated from CH030 (Δ*eptA*) grown in Fe^3+^ produced a peak at m/z 1928.8 atomic mass units (amu) indicative of L-Ara4N modification (Fig. 4B). Whereas lipid A from CH040 (Δ*arnT*) produced major peaks at m/z 1920.8 and 2043.5 amu corresponding to the predicted pEtN modified species (Fig. 4C). The 1-PP species at m/z 1876.9 amu produced by wild-type grown in the absence of iron (Fig. 4A) was not detected upon Fe^3+^ induction (data not shown). Similar lipid A profiles were obtained in N-minimal medium containing 10 mM Mg^2+^, at pH 5.8 (Fig. 3B). Interestingly, LpxT-dependent lipid A modification was not recovered to wild-type levels in *eptA* or *arnT* mutants. Only in the double mutant CH034 (Δ*arnT*Δ*eptA*) could any 1-PP lipid A be detected (Fig. 3, lane 5) and was only barely detectable, therefore no considerable LpxT activity could be recovered even if other phosphate modifying enzymes were inactivated. In the absence of Fe^3+^ induction, the lipid A profiles of CH030 (Δ*arnT*), CH040 (Δ*eptA*) and the double mutant CH034 (Δ*arnT*Δ*eptA*) were similar to that of wild-type (data not shown).

The lipid A profile of *eptA* and *arnT* mutants was also analysed in the WD101 (*pmrA*^c^) background (Fig. S1). Again, 1-PP-lipid A was not recovered to wild-type levels in the *pmrA*^c^ background even in strain WD*arnTeptA* (lane 5). Moreover, single and double pEtN-lipid A species were highly induced compared with L-Ara4N-lipid A substitutions (Fig. S1), suggesting a dominant EptA-dependent lipid A modification in an *E. coli pmrA*^c^ background. Together, these results confirm that ArnT or EptA are not essential for LpxT regulation. However, when PmrA is induced or constitutively expressed, LpxT-dependent lipid A modification is lost, suggesting that PmrA may play a role in *lpxT* regulation.

### PmrA is not involved in transcription of LpxT

Our next approach was to determine if PmrA served as a transcriptional regulator of *lpxT*. In *E. coli* K12, the *lpxT* (*yeiU*) gene is downstream of the *yeiR* gene separated by only 38 bp. To elucidate whether both genes are transcribed into the same mRNA, *yeiR* and *lpxT* transcripts were evaluated by reverse transcription-PCR (Fig. S2). A PCR product as a single transcript of both genes using cDNA W3110 template was not achieved. These results confirmed that *lpxT* and *yeiR* are transcribed independently (Fig. S2).

**Fig. 5:**
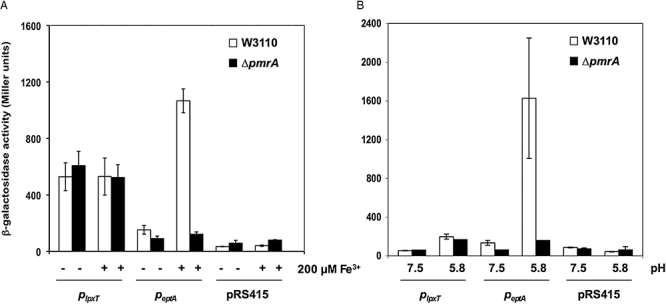
Transcriptional regulation of *lpxT* gene is PmrA-independent. Promoter activation was evaluated by β-galactosidase activity in strains W3110 (wild-type) and mutant CH020 (Δ*pmrA*) harbouring plasmids pMST2 (*p_lpxT_*::pRS415), pCH02 (*p_eptA_*::pRS415) or pRS415. Cells were grown in (A) LB medium either with or without 200 µM FeSO_4_, or in (B) N-minimal medium supplemented with 10 mM MgCl_2_ at pH 5.8 or 7.5. Cells were harvested at OD_600_ 0.5–0.6 followed by β-galactosidase assay. The data show the mean and standard deviation of three independent repetitions.

To perform β-galactosidase assays, the 250 bp upstream region of *lpxT* was cloned into a promoter-less plasmid pRS415 carrying the *lacZ* reporter gene. Since *Salmonella* EptA (PmrC) was reported to be under PmrA-regulation (Lee *et al*., 2004; Tamayo *et al*., 2005b), a 300 bp upstream region of *E. coli eptA* cloned into pRS415 was also evaluated. W3110 and its isogenic strain CH020 (Δ*pmrA*) harbouring these plasmids were grown in LB medium supplemented with or without 200 µM FeSO_4_ (Fig. 5A). Our results indicate that *p_lpxT_* (*lpxT* promoter) was not upregulated in the presence of Fe^3+^. Furthermore, W3110 and CH020 (Δ*pmrA*) did not exhibit differences in *p_lpxT_* activation. Contrary, *p_eptA_* (*eptA* promoter) was induced sevenfold in the presence of Fe^3+^, and this induction was lost in strain CH020 (Δ*pmrA*). These data confirmed that *p_eptA_* was activated in a PmrA-dependent manner. Background activity from plasmid pRS415 was limited to 50–80 units or lower in the negative controls, which is consistent with the reported plasmid background (Simons *et al*., 1987). Activation of *p_lpxT_* was also evaluated in N-minimal medium containing 10 mM Mg^2+^, at pH 5.8 or 7.5 (Fig. 5B). Again, induction of *p_lpxT_* was PmrA-independent. As we expected, PmrA-dependent *p_eptA_* was activated displaying 12-fold more activity at pH 5.8 compared with pH 7.5 in W3110 strain. Furthermore, *lpxT* transcription was evaluated quantitatively by real-time PCR. The amount of *lpxT* transcript did not show statistical differences in W3110 and WD101 (*pmrA^C^*) strains when grown in LB medium (data not shown). These results confirm that PmrA does not regulate *lpxT* transcription.

### LpxT-dependent lipid A modification is regulated post-translationally

**Fig. 6:**
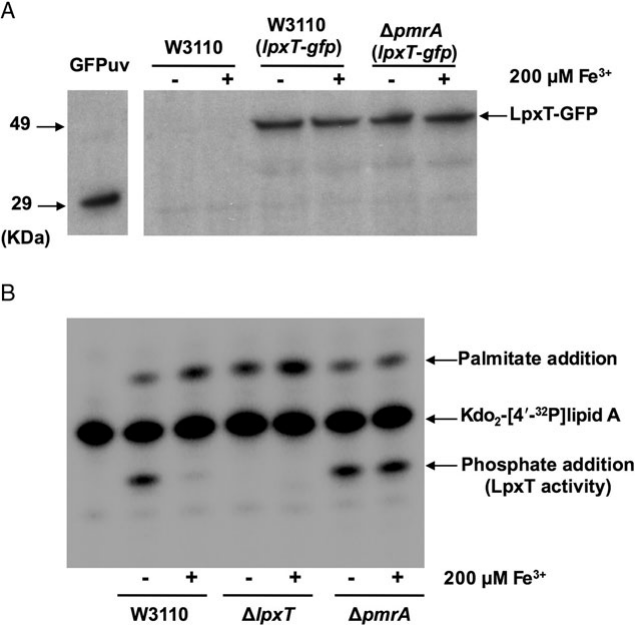
PmrA regulates LpxT-dependent lipid A modification in the periplasm. A. Western blot showing that LpxT–GFP protein is synthesized and assembled into the membrane in a PmrA-independent manner. Strains W3110, CH01 (W3110, *lpxT–gfp*) and CH021 (Δ*pmrA*, *lpxT–gfp*) were grown in LB medium with 200 µM or without FeSO_4_. Membrane protein fractions (5 µg) and cytoplasmic GFP_uv_ protein (2 µg) were used for western analysis. Soluble GFPuv and W3110 membranes served as positive and negative controls respectively. Analysis of the soluble fraction demonstrated that LpxT–GFP was only present in the membrane fraction (see Fig. S4). Arrows indicate the molecular mass (kDa) of protein standards. B. Enzymatic assay for phosphate addition to Kdo_2_-[4′-^32^P]-lipid A indicates inhibition of LpxT within the membrane following PmrA activation. Membrane samples from W3110, MST01 (Δ*lpxT*) and CH020 (Δ*pmrA*) grown in the presence or absence of Fe^3+^ were used as the enzyme source. Assays were performed as described in the Material and Methods. The reaction products were separated by TLC and detected by phosphorimaging. Palmitate addition catalysed by PagP has also been indicated.

Although PmrA does not regulate *lpxT* at the transcriptional level, growth conditions promoting PmrA activation result in inhibition of LpxT-dependent phosphorylation of lipid A in whole cells. To determine if LpxT is successfully assembled into the membrane, we monitored the expression of a chromosomal *lpxT–gfp* fusion protein under the control of its native promoter in strain CH01. The fusion protein was also expressed in strain CH021 (Δ*pmrA, lpxT–gfp*). LpxT–GFP was fully functional in whole cells as indicated by the production of 1-PP lipid A (Fig. S3). Presence of LpxT–GFP in membranes from cells grown with or without 200 µM FeSO_4_ was evaluated by Western blotting using anti-GFP antibody (Fig. 6A). A 29.3 kDa GFPuv protein was used as positive control for anti-GFP polyclonal antibody, whereas W3110 wild-type was considered as negative control. Strains CH01 and CH021 synthesized and assembled LpxT–GFP into the membrane even in the presence of Fe^3+^. LpxT–GFP was not found in the soluble fraction (Fig. S4), which was expected given that LpxT contains six transmembrane domains. The protein molecular mass calculated for LpxT–GFP fusion was 53.4 kDa but a 49 kDa protein was observed (Fig. 6A). The gel shifting in SDS-PAGE gel migration is commonly observed in membrane proteins (El Ghachi *et al*., 2004; Rath *et al*., 2009). Thus, Fe^3+^ induction of PmrA does not interfere with the synthesis or assembly of LpxT into the bacterial membrane.

**Fig. 10:**
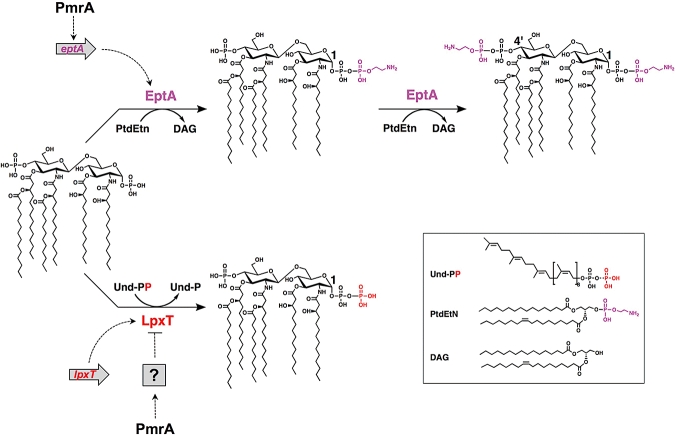
Lipid A modification reactions catalysed by LpxT and EptA. LpxT transfers a phosphate group (red) from undecaprenyl pyrophosphate to the 1-position of lipid A. Specific environmental stimuli, such as exposure to high [Fe^3+^] or reduction in extracellular pH, results in (i) the post-translational inactivation of LpxT and (ii) activation of the transcriptional regulator PmrA. PmrA-activation leads to increased expression of EptA that competes for the 1-position of lipid A. Presumably, EptA utilizes phosphatidylethanolamine (PtdEtn) as the pEtN (magenta) donor for lipid A modification generating diacylglycerol (DAG). The latter is supported by the previous finding that PmrA^c^*E. coli* are unable to modify their lipid A with pEtN, in a PtdEtN deficient background (Trent *et al*., 2001b). Modification at the 1-position is followed by a second modification at the 4′-position of lipid A promoting polymyxin resistance.

Given that PmrA does not inhibit localization of LpxT to the membrane, we determined whether or not LpxT activity was inhibited under PmrA activating conditions using an *in vitro* assay system. For this purpose, membranes of W3110, MST01 (Δ*lpxT*) and CH020 (Δ*pmrA*) strains were prepared from cultures grown in LB medium with or without 200 µM FeSO_4_. Phosphorylation of lipid A by LpxT was evaluated using [4′-^32^P]Kdo_2_-lipid A (Fig. 1A) as the acceptor substrate (Fig. 6B) and endogenous undecaprenyl-pyrophosphate (Fig. 10, inset) within the membrane fraction served as the phosphate donor. As expected, membranes isolated from Δ*lpxT* showed no phosphotransferase activity. Wild-type membranes catalysed the addition of phosphate to [4′-^32^P]Kdo_2_-lipid A, whereas phosphotransferase activity was only barely detectable in membranes from cells grown in the presence of Fe^3+^. Interestingly, membranes of mutant CH020 (Δ*pmrA*) catalysed phosphate addition to [4′-^32^P]Kdo_2_-lipid A even under Fe^3+^ inducing conditions (Fig. 6B). Therefore, although the expression of LpxT is not PmrA-regulated, in the presence of the inducing signal (i.e. Fe^3+^) PmrA is necessary for the loss of LpxT activity in the bacterial membrane. A more hydrophobic reaction product resulting from the PagP catalysed transfer of palmitate (Fig. 1B) was detected in all assays (Fig. 6B) (Bishop *et al*., 2000).

### LpxT and EptA compete for the modification of lipid A

**Fig. 7:**
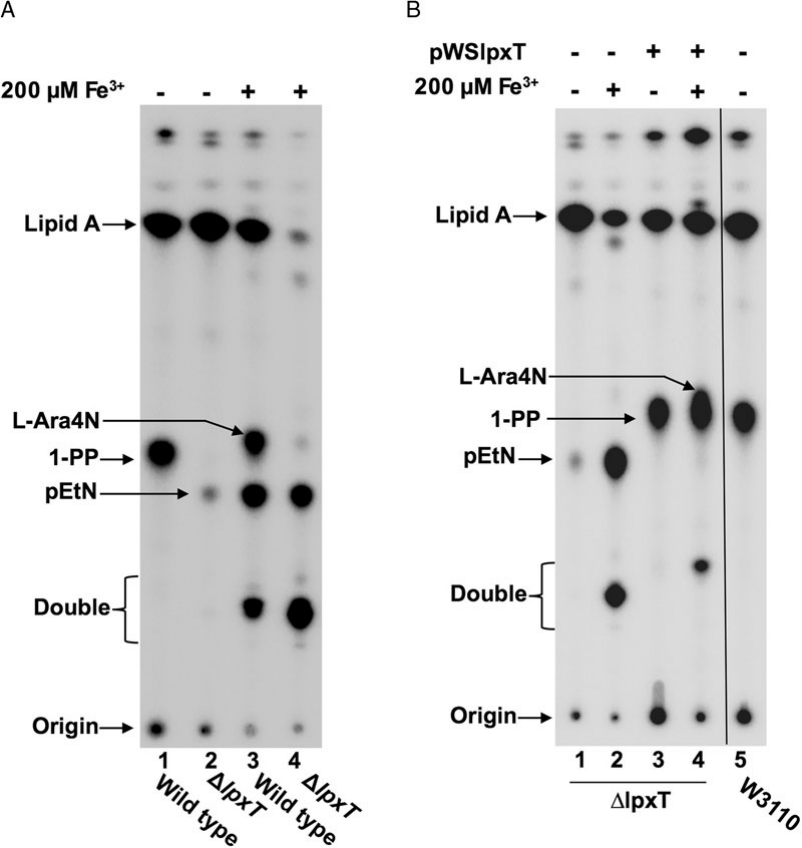
EptA-dependent lipid A modification is induced in an *lpxT*-deficient mutant. A. Comparative analysis of ^32^P-lipid A species produced by strains W3110 and MST01 (Δ*lpxT*) grown in the presence or absence of Fe^3+^. B. ^32^P-lipid A profile of Δ*lpxT* (MST01) expressing LpxT *in trans* grown in the presence or absence of Fe^3+^. W3110 grown in LB medium was used as a positive control for 1-PP synthesis. Major lipid A species are indicated as in Fig. 2.

As we reported previously (Touze *et al*., 2008), based upon TLC analysis the deletion of *lpxT* promotes a basal level of *E. coli* EptA activity independent of Fe^3+^ induction (Fig. 7A, lane 2). This result was confirmed here by MALDI-TOF mass spectrometry. Loss of *lpxT* resulted in the production of major peaks at m/z 1796.8 and 1920.6 amu (Fig. 4D) corresponding to 1,4′-*bis*-phosphorylated and pEtN modified lipid A. Interestingly, when strain MST01 (Δ*lpxT*) was grown under Fe^3+^ inducing conditions there was an unexpected increase in pEtN modification of lipid A with a concomitant loss of L-Ara4N modification compared with wild-type (Fig. 7A, lane 4). Thus, although LpxT-dependent phosphorylation of lipid A disappears upon activation of PmrA, the presence of LpxT in the membrane directly affects the ratio of pEtN and L-Ara4N modified species in whole cells. We were curious if overexpression of *lpxT in trans* could decrease EptA modification during PmrA activation. Hence, the lipid A profiles of strain MST01 (Δ*lpxT*) expressing pWSK29 plasmid-born *lpxT* were evaluated (Fig. 7B). LpxT expressed *in trans* restored the synthesis of 1-PP lipid A and inhibited basal EptA activity (i.e. wild-type phenotype was recovered) (Fig. 7B, lane 3). Fe^3+^ induction promoted the L-Ara4N-lipid A synthesis in the same strain, as we can observe a shift in the spot where 1-PP and L-Ara4N lipid A are co-migrating (lane 4). On the other hand, pEtN addition is lost upon introduction of plasmid born LpxT even under Fe^3+^ inducing conditions. Together, these results suggest that even though LpxT activity is inhibited during PmrA activation it is possible to overcome this inhibition by overexpression of the protein.

**Fig. 8:**
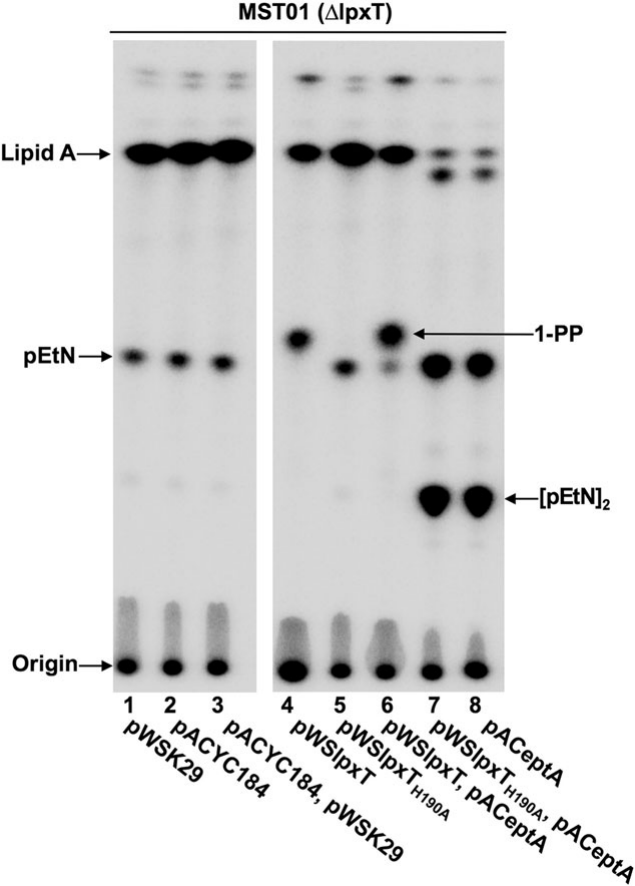
Simultaneous *in trans* expression of EptA and LpxT results in decreased pEtN modification. ^32^P-lipid A was isolated from strain Δ*lpxT* (MST01) grown in LB harbouring the indicated plasmids. Deletion of *lpxT* results in the production of a minor species of lipid A containing a single pEtN residue (Fig. 4D). The latter is observed in MST01 harbouring control plasmids (pWSK29 and pACYC94). Lipid A species were analysed by TLC and visualized by phosphorimaging. Major lipid A species are indicated as in Fig. 2 with modification of lipid A with two pEtN residues indicated by [pEtN]_2_.

EptA and LpxT are both inner membrane proteins with their active sites located in the periplasmic compartment (Lee *et al*., 2004; Tatar *et al*., 2007; Touze *et al*., 2008). LpxT is a member of the phosphatidic acid-phosphatase family (PAP2) characterized by a conserved phosphatase motif KX_6_RP-(X_12–54_)-PSGH-(X_31–54_)-SRX_5_HX_3_D (Stukey and Carman, 1997). To evaluate whether inhibition of EptA modification is directly affected by LpxT phosphotransferase activity, residue His-190 (PSGH_190_) within the predicted active site of LpxT (Tatar *et al*., 2007) was replaced with an alanine (H190A). LpxT (LpxT_WT_) and the H190A (LpxT_H190A_) mutant were expressed in plasmid pWSK29. EptA was expressed in plasmid pACYC184 (pACeptA) avoiding plasmid incompatibility. Expression of LpxT_H190A_*in trans* was unable to complement the loss of 1-PP in strain MST01 (Δ*lpxT*) (Fig. 8, lane 5) as compared with LpxT_WT_ (lane 4). Coexpression of LpxT_WT_ and EptA produced both 1-PP and pEtN-lipid A species, with LpxT_WT_ clearly the predominate activity (lane 6). In contrast, coexpression of LpxT_H190A_ and EptA led to extensive double pEtN modification (lane 7) identical to that seen when EptA was expressed alone (lane 8). The latter result was confirmed by mass spectrometry (data not shown) yielding similar results to those shown in Fig. 4C. In summary, these data demonstrate that LpxT and EptA compete for modification of the 1-phosphate group of lipid A. Furthermore, substitution with pEtN at both phosphate groups occurs only in the absence of LpxT, suggesting that pEtN addition occurs in a sequential fashion with modification of the 1-phosphate group followed by modification at the 4′-position.

### EptA-dependent lipid A modification is required for resistance to polymyxin B

Polymyxin B is a cyclic amphipatic peptide that binds to the lipid A anchor of LPS eventually leading to disruption of the bacterial membrane. Gram-negative microorganisms respond to attack by antimicrobial peptides by modifying their lipid A (Gunn *et al*., 2000) promoting resistance. We investigated what type of PmrA-dependent lipid A modifications in *E. coli* plays a role in polymyxin resistance in a *pmrA^c^* background using strain WD101. The minimal inhibitory concentrations (MICs) were determined on LB agar using ETest polymyxin B strips (Table 1). WD101 (*pmrA*^c^) was 40-fold more resistant to polymyxin compared with its isogenic parent, W3110. Loss of *arnT*, which still allows for the synthesis of single and double pEtN modifications (Fig. S1), showed an eightfold decrease in resistance, whereas *eptA* mutants showed a 20-fold decrease. Therefore, in *E. coli*, EptA plays a dominant role in polymyxin resistance. A double mutant (WDeptAarnT) unable to modify its lipid A with either L-Ara4N or pEtN was slightly more resistant (MIC, 0.60 ± 0.05 µg ml^−1^) than wild-type *E. coli* (MIC, 0.30 ± 0.05 µg ml^−1^) (Table 1). This slight difference may arise because LpxT activity is present in wild-type W3110 contributing to the overall negative charge of the bacterial surface, whereas in WDeptAarnT 1-PP lipid A is nearly absent (Fig. S1). Finally, overexpression of LpxT *in trans* in WD101 resulted in loss of pEtN modification (Fig. S5) and compromised WD101 polymyxin resistance (Table 1). MICs were also determined in LB broth (data not shown) and were found to be similar to the agar based assays (Table 1).

**Table 1:** Polymyxin minimal inhibitory concentration (MIC) of *E. coli* WD101 (*pmrA^C^*) strains.

Strain	Plasmid	Polymyxin B MIC (µg ml^−1^)
W3110	–	0.30 ± 0.05
WD101	–	12.5 ± 2.50
WDeptA	–	0.60 ± 0.10
WDarnT	–	1.50 ± 0.50
WDeptAarnT	–	0.60 ± 0.05
WD101	pWSlpxT	4.70 ± 1.20
WDarnT	pWSlpxT	0.90 ± 0.14

### Deletion of lpxT increases EptA-dependent lipid A modification in *Salmonella*

**Fig. 9:**
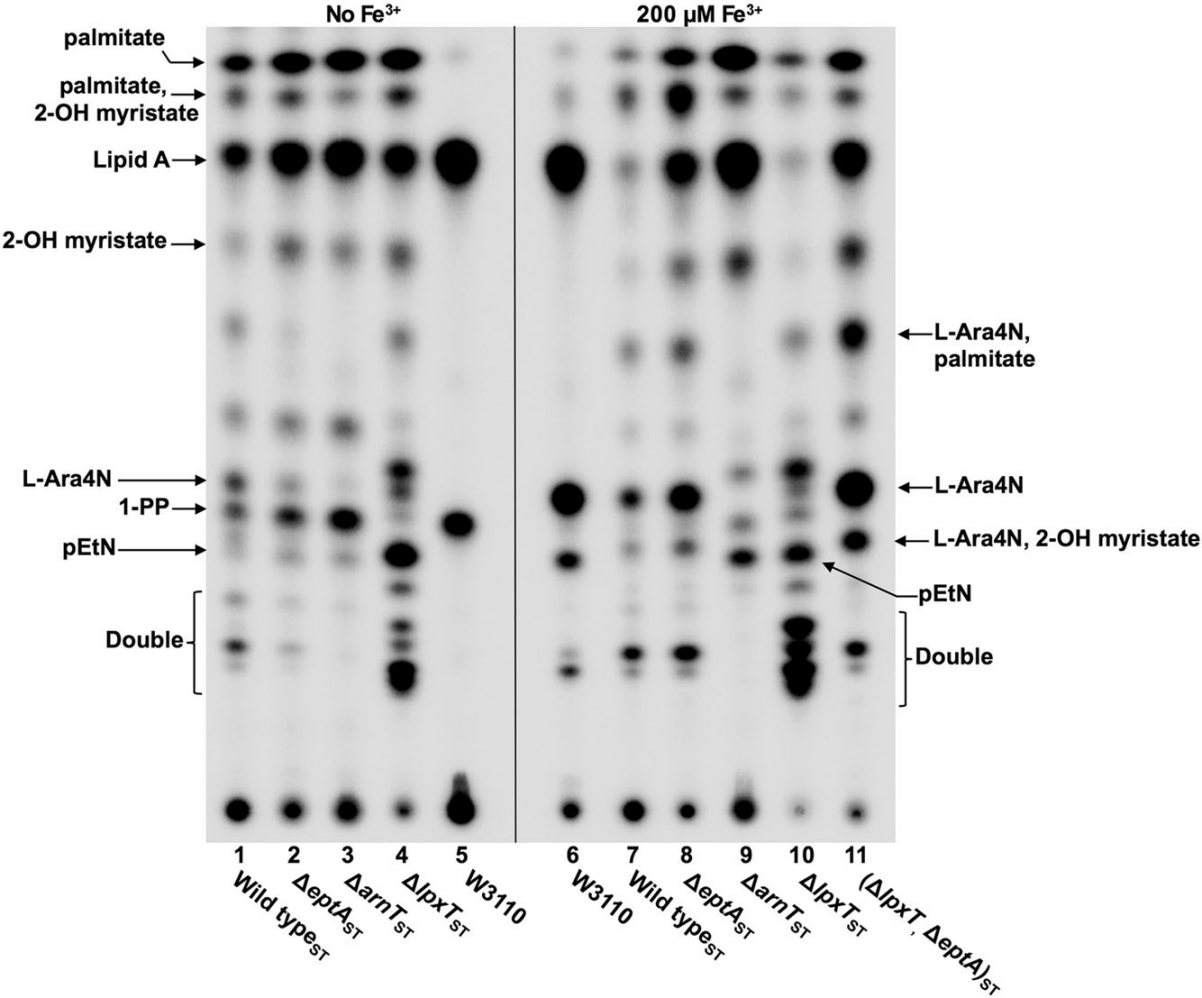
Loss of LpxT results in increased pEtN modification in *Salmonella*. Comparative analysis of ^32^P-lipid A species produced by *S. typhimurium* LT2 strains harbouring mutations in lipid A modification enzymes grown in the presence or absence of Fe^3+^. ^32^P-lipid A from W3110 was used as reference for the migration of modified forms of lipid A. The major lipid A species are indicated as in Figure 2. Additional modifications occurring in *Salmonella* including LpxO catalysed hydroxylation producing lipid A with 2-OH myristate and increased palmitate addition by PagP (Gibbons *et al*., 2000) are indicated where necessary for clarity.

Since LpxT activity appears to compete with pEtN modification in *E. coli*, we performed a comparative analysis of the lipid A profiles of *Salmonella typhimurium* LT2 (wild-type_ST_) and its isogenic mutant CH05 (Δ*lpxT_ST_*) (Fig. 9). In *Salmonella*, the presence of additional lipid A modifying enzymes results in a more complex lipid A profile. LpxO, an inner membrane dioxygenase that is not present in *E. coli*, hydroxylates the secondary myristate at the 2′-position of *Salmonella* lipid A (Gibbons *et al*., 2000). Also, PagP-dependent palmitoylation occurs more frequently in *Salmonella* and L-Ara4N addition is more prevalent than pEtN modification (Zhou *et al*., 2001). To aid in identification of pEtN and L-Ara4N modified species, lipid A isolated from W3110 grown in Fe^3+^ was included as a control.

In wild-type_ST_, both 1-PP and L-Ara4N modified lipid A were easily detected, whereas pEtN modification was less evident (Fig. 9, lane 1). Upon deletion of *lpxT_ST_* (strain CH05) there was a drastic increase in lipid A modifications even in the absence of iron induction with pEtN modification predominating (Fig. 9, lane 4). Fe^3+^ induction promoted L-Ara4N modification and inhibited LpxT modification in wild-type LT2 (lane 7), whereas in strain CH05 (Δ*lpxT_ST_*) pEtN modification was again increased (lane 10). The identification of *Salmonella* lipid A species was based upon previous reports (Zhou *et al*., 1999; 2000; 2001;) and confirmed by generating strains CH06 (Δ*arnT_ST_*), CH07 (Δ*eptA_ST_*), and CH057 (Δ*lpxT_ST_*, Δ*eptA_ST_*). The doubly modified lipid A species prevalent in the Δ*lpxT_ST_* background during Fe^3+^ induction were lost upon introduction of Δ*eptA_ST_* (lane 11). On the other hand, deletion of *eptA* in the wild-type background had little effect on the lipid A profile (lane 8). Here again, these data support a role for LpxT in the reduction of EptA activity. Furthermore, loss of functional *Salmonella* LpxT results in a shift from lipid A species primarily modified with L-Ara4N to modification with pEtN.

## Discussion

Remodelling of the lipid A component of Gram-negative bacteria is one example of how Gram-negative organisms respond to their surrounding environment. In *E. coli* and *Salmonella*, the PmrA–PmrB and PhoP–PhoQ two-component systems are primarily responsible for regulating the expression of the molecular machinery required for the structural modification of LPS. PmrA controls synthesis of both ArnT and EptA, two inner membrane enzymes that modify the phosphate groups with amine-containing substituents by the addition of L-Ara4N (Trent *et al*., 2001a) and pEtN (Trent and Raetz 2002; Lee *et al*., 2004) respectively. Different studies have described ArnT and EptA-dependent lipid A modifications in response to signals such as vanadate, low pH, low Mg^+2^, Al^3+^, or high Fe^3+^, which are all PmrA–PmrB mediated (Zhou *et al*., 1999; Gibbons *et al*., 2005; Nishino *et al*., 2006).

A third type of lipid A modification is carried out by LpxT, which adds a phosphate specifically at the 1-position of lipid A generating a 1-diphosphate species (1-PP) and increasing the overall negative charge of the bacterial surface (Touze *et al*., 2008). Interestingly, LpxT utilizes undecaprenyl pyrophosphate (Und-PP) as its phosphate donor contributing to the cellular pool of undecaprenyl phosphate (Und-P). The latter serves as an essential carrier lipid for the transport of glycan intermediates across the membrane in the synthesis of cell wall polysaccharides, such as O-antigen and peptidoglycan. Contrary to ArnT and EptA, how LpxT-dependent lipid A modification is regulated was not clear. Zhou and co-workers (Zhou *et al*., 2001) suggested that lipid A decorated with pEtN and L-Ara4N moieties are not compatible with formation of 1-diphosphate lipid A. However, the role of PmrA on LpxT regulation was not established. Our studies presented here demonstrate that PmrA-dependent lipid A modification impedes decoration of lipid A by LpxT even in the absence of ArnT or EptA. Given the wide range of signals that can induce the PmrA–PmrB two-component system, we focused on iron.

Due to the inverse relationship of PmrA and 1-PP lipid A formation, we expected that *lpxT* transcription was negatively regulated by PmrA. However, activation of PmrA had no effect on *lpxT* promoter activity during β-galactosidase reporter assays (Fig. 5). Furthermore, the levels of *lpxT* transcript as determined by quantitative PCR (data not shown) were the same in both wild-type (W3110) and PmrA^c^ (WD101) strains. These data are supported further by microarray analysis in *pmrA*^c^ and *pmrA* null mutants where levels of *lpxT* transcripts did not change in cells grown in 400 µM Fe^3+^-containing LB medium (Hagiwara *et al*., 2004). This led us to explore whether or not LpxT activity was regulated after its insertion into the membrane. First, detection of an LpxT–GFP recombinant protein revealed that PmrA-dependent regulation occurs following LpxT assembly into the membrane (Fig. 6A). Second, although PmrA-activated cells synthesize LpxT, its activity is inhibited during *in vitro* phosphotransferase assays. This activity was restored in membranes isolated from a PmrA-deficient mutant grown in Fe^3+^, confirming a post-translational inhibition of LpxT (Fig. 6B).

The current work does, however, clearly demonstrate that pEtN addition is only efficient in the absence of LpxT (Figs 7 and 8). Cells expressing LpxT and EptA at similar copy number preferentially produce 1-PP lipid A, whereas pEtN addition is inhibited (Fig. 8). PmrA-induced expression of EptA may help in overcoming cellular levels of LpxT; however, this cannot be the sole reason for decreased 1-PP modification since LpxT activity is not restored in *eptA, arnT* double mutants (Fig. 3 and Fig. S1). Therefore, in order to promote successful pEtN modification, LpxT activity must be directly inhibited in PmrA-activated cells (Fig. 10) allowing for the sequential addition of pEtN to the 1- and 4′-phosphate groups. The PmrA–PmrB two-component system is involved in regulation of a wide number of genes (Gunn, 2008). We anticipate that a non-identified protein plays a direct role in the regulation of LpxT activity after the bacterium sense changes in the surrounding environment. Given our current results and the fact that the LpxT active site is on the periplasmic face of the inner membrane, this regulation most likely occurs within the periplasm. Perhaps LpxT is sequestered within the membrane away from the lipid A anchor of LPS or actively involved in the modification of a second, as yet unidentified, target. Given the importance of bacterial surface modifications, how LpxT is regulated within the bacterial membrane remains under investigation.

During activation of *E. coli* PmrA, pEtN modification of lipid A phosphate groups is predominant. For example, in mutants lacking *arnT* double pEtN modifications are easily detectable (Figs 3 and 4; Fig. S1), but species bearing two L-Ara4N residues were not. The opposite scenario can be found in wild-type *S. typhimurim* LT2 where L-Ara4N modified lipid A species are prevalent (Zhou *et al*., 2000; Zhou *et al*., 2001) (Fig. 9). Interestingly, in CH05 (Δ*lpxT*_ST_) L-Ara4N addition drastically decreases and pEtN modification predominates, indicating the ability of LpxT to inhibit EptA modification in *Salmonella* as well. Previously, it was shown that EptA (also known as PmrC) did not play any significant role in providing resistance to CAMPs in *S. typhimurium* (Lee *et al*., 2004). Contrary to this observation, we demonstrate that EptA is critical for CAMP resistance in *E. coli*. Based upon the detailed analysis presented here, both observations are correct.

This report emphasizes the importance of a well-defined regulation of lipid A modification systems within the bacterial membrane. In response to the surrounding environment, phosphorylation of lipid A by LpxT must be inhibited to allow EptA-dependent modification, thereby avoiding deleterious effects to the bacterium (e.g. CAMP attack). Other membrane phosphatases are dedicated to the hydrolysis of Und-PP (El Ghachi *et al*., 2004; 2005;), and so, LpxT is not essential for Und-P synthesis. However, this finding demonstrated the utility of Und-PP as a high-energy phosphate donor opening the possibility that other unidentified bacterial components are phosphorylated within the periplasm. Perhaps, this is why LpxT expression occurs independently of 1-PP modification. The fact that EptA modifies lipid A under non-inducing PmrA conditions indicates a basal level of expression opening the possibility that EptA may also participate in other cellular processes. For example, our laboratory recently demonstrated that an EptA homologue in *Campylobacter jejuni* not only modifies lipid A, but also transfers a pEtN residue to the flagellar rod, promoting its assembly and motility (Cullen and Trent, 2010). A proteomic study identified *E. coli* EptA in complex with ZipA, an essential component of the septal ring, suggesting a role for EptA in cell division (Stenberg *et al*., 2005). Like LpxT, *E. coli* EptA may transfer pEtN to as yet unidentified targets within the periplasm. For these reasons, it is important to pursue a deeper understanding of lipid A modification systems.

## Experimental procedures

### Strains, media and growth conditions

All relevant strains are listed in Table S1. *E. coli* K-12 strains were grown at 37°C on LB broth or agar (DIFCO), or N-minimal medium containing 0.1% casamino acids and 38 mM glycerol (Chamnongpol *et al*., 2002). N-minimal medium was buffered with 0.1 M Bis-Tris, adjusted to pH 5.8 or 7.5. In experiments for which FeSO_4_ or MgCl_2_ was added to the media, bacteria were first grown overnight in LB and washed in the same medium to be cultured absent of additional metals. Bacteria were then diluted to OD_600_ 0.05 in fresh media containing the indicated concentrations of FeSO_4_ or MgCl_2_. Antibiotics ampicillin (100 µg ml^−1^), kanamycin (30 µg ml^−1^), chloramphenicol (30 µg ml^−1^) and polymyxin B were used where appropriate.

### Standard DNA and RNA methods

Genomic DNA was extracted with Easy DNA Kit (Invitrogen). Plasmid DNA was purified with QIAGEN Spin Prep Kit (Stratagene). DNA fragments were amplified using *PfuTurbo^R^* DNA polymerase (Stratagene) or Takara *Ex Taq* DNA polymerase (Takara). PCR products were isolated from agarose gels using QIAquick PCR gel extraction kit. Primers were purchased from Invitrogen. All restriction endonucleases and T4 DNA ligase were purchased from New England Biolab. Total RNA was extracted with Trizol Max bacterial RNA isolation kit (Invitrogen) from cells grown in LB up to an OD_600_ of 1.0. RNA was treated with RQ1 RNAse-free DNAse (Promega) to eliminate DNA contamination. RNA quality was evaluated by gel electrophoresis (Sambrook and Russell, 2001). cDNA synthesis was performed according to Verso cDNA Kit (ThermoFisher Scientific).

### Construction of chromosomal gene deletion mutants

Chromosomal in-frame gene deletions were generated in the DY330 strain based on λ Red recombinase system (Datsenko and Wanner, 2000). Design of primers for linear cassette was based on a 20 bp target region in a plasmid template pKD3 or pKD4 for resistance cassette and a 50 bp homologous to chromosomal region flanking the gene of interest, including the start codon and at least three codons before the stop codon of the gene. Specific linear DNA cassettes were obtained using the appropriate primers listed in Table S2. The generated PCR products were digested with DpnI for 1 h at 37°C, purified and used for electro-transformation of induced DY330 cells. Induction of λ prophage recombination activity and preparation of competent cells were performed as described previously (Yu *et al*., 2000). Candidate mutants were selected on LB agar containing appropriate antibiotics. P1 *vir* phage transduction was used to move selectable deleted genes from the donor DY330 strain to the recipient W3110 (Sambrook and Russell, 2001). Mutations were confirmed by PCR using primers (Table S2) annealing outside of flanking regions of deleted gene. When necessary, excision of resistance gene was carried out using the helper plasmid pCP20 (Datsenko and Wanner, 2000).

Chromosomal gene deletions in *S. typhimurium* were based on λ Red recombinase system (Datsenko and Wanner, 2000). Cells carrying the red helper plasmid pKD46 were induced with 1 mM l-arabinose and competent cells were prepared as described elsewhere (Gerlach *et al*., 2007). Linear DNA cassettes were obtained with primers designed to amplify genes encoding resistance cassettes (Table S2) carried out by plasmids pKD3 or pKD4 (Table S1), following the same strategy described above for *E. coli*. Induced competent *S. typhimurium* cells were transformed with linear PCR products and proper insertion was verified by PCR using specific primers (Table S2). The temperature sensitive plasmid pKD46 was cured from mutant strains by growing on LB agar at 42°C for 14 h. When necessary P22 phage transduction was used to move selectable deletions. Furthermore, excision of resistance cassettes was carried out using the helper plasmid pCP20 (Datsenko and Wanner, 2000).

### Generation of chromosomal *lpxT–gfp* fusion

The GFP_mut3_ protein was tagged to the C-terminal region of LpxT protein. For this purpose, *gfp_mut3_* and *nptII* genes were amplified as a single PCR product using the primers LpxTgfpF and LpxTgfpR (Table S2) and the plasmid p3174 (Gerlach *et al*., 2007) as DNA template. Primer LpxTgfpF contains 50 bp upstream of *lpxT* stop codon and 21 bp of reporter gene while primer LpxgfpR has 50 bp downstream of lpxT stop codon and 21 bp homologous region to plasmid p3174. The resulting 2.2 kb linear *gfpmut3–nptII* cassette was used for electro-transformation of induced DY330 cells, following a similar strategy for chromosomal gene deletion described above. P1 *vir* transduction was used to move the reporter gene fusion into recipient strains. Chromosomal integration of *gfp_mut3–_nptII* reporter cassette into *lpxT* gene was verified by sequencing using primers UlpxT and GFP-Fu independently (Table S2).

### Plasmid constructs

Constructs for membrane protein expression was performed using the low-copy-number plasmid pWSK29 (Wang and Kushner, 1991). The *lpxT* gene was amplified using primers LpxTF and LpxTR (Table S2), digested with EcoRI and BamHI, and ligated into plasmid pWSK29 yielding pWSlpxT. The *eptA* gene was amplified using primers 50eptAF and 50eptAR (Table S2), digested with HindIII and BamHI, and ligated into plasmid pACYC184 yielding pACeptA. LacZ reporter fusions were constructed as follows. A 250 bp fragment corresponding to the upstream region of *lpxT* gene was amplified using primers PlpxTF and PlpxTR (Table S2). A second 300 bp amplicon, corresponding to the upstream region of *eptA* gene, was obtained using primers PeptAF and PeptAR (Table S2). Each amplicon was digested with BamHI and EcoRI restriction enzymes and ligated into the MCS of promoter-less plasmid pRS415, generating plasmids pMST2 and pCH02 respectively. All constructs were verified by sequencing.

### Site-directed mutagenesis of lpxT

Site-directed mutagenesis of *lpxT* was carried out using the QuickChange site-directed mutagenesis kit (Stratagene) according to the manufacturer's instructions with plasmid pWSlpxT as the template. Primers LpxTA190-S and LpxTA190-A were used to mutate amino acid H190 to A190 resulting in plasmid pWSlpxT_H190A_.

### Immunoblotting analysis of YeiU-GFP protein

Cells expressing the YeiU-GFP fusion protein were grown in LB with or without 200 µM FeSO_4_. Membrane pellets from cells lysed by French Press were homogenized and washed twice as previously described (Trent *et al*., 2001c) using 50 mM HEPES pH 7.0 buffer to ensure complete removal of cytoplasmic components. Similarly, the soluble fractions were cleared twice of membrane components as previously described (Trent *et al*., 2001c). The GFPuv protein was isolated from the cytoplasm fraction following the Sambrook protocol (Sambrook and Russell, 2001) and was used as a positive control for immunoblotting detection. Protein concentration was determined by BCA method kit (Pierce). Proteins (5 µg) from the membrane fraction were incubated at 42°C for 50 min and resolved on 4–12% NUPAGE Bis-Tris gels (Invitrogen) under reducing conditions. Proteins were transferred to nitrocellulose membranes as described by the manufacturer using XCell II blot module (Invitrogen). Immunodetection was performed using 1:2000 dilution of rabbit Anti-GFP (Molecular Probes, Invitrogen) as the primary antibody and anti-rabbit IgG horseradish peroxidase-linked as the secondary antibody (Amersham). Chemoluminiscence immunodetection was carried using the ECL kit (Amersham).

### Isolation of labelled Lipid A

Cultures were diluted to an OD_600_ of 0.05 in 5 ml of fresh medium and labelled with 2.5 µCi ml^−1^^32^P_i_ (Amersham) in LB broth or N-minimal media as indicated. Cells were harvested at an OD_600_ of 1.0 by centrifugation and the isolation of ^32^P-labelled lipid A carried out by mild acid hydrolysis as previously described (Zhou *et al*., 1999; Trent *et al*., 2001a). ^32^P-lipid A species (∼10 000 cpm·per lane) were analysed by TLC in a solvent system of chloroform, pyridine, 88% formic acid and water (50:50:16:5, v/v) and visualized by phosphorimaging analysis (Bio-Rad PMI).

### Assay for LpxT catalysed phosphate addition to Kdo_2_-[4′-^32^P]lipid A

Double-washed membrane fractions from cultures harvested at OD_600_ 1.0 served as the enzyme source. Membrane pellets were homogenized and washed twice as previously described (Trent *et al*., 2001c), using 50 mM HEPES pH 7.0 buffer to ensure complete removal of cytoplasmic components. Radiolabelled Kdo_2_-[4′-^32^P]lipid A was prepared as previously described and served as the lipid acceptor (Tran *et al*., 2004). LpxT phosphotransferase activity was evaluated in a 10 µl reaction containing 5 µg of membrane protein, 50 mM HEPES pH 7.0, 0.2% Triton X-100, and 2.5 µM Kdo_2_-[4′-^32^P]lipid A (∼5000 cpm nmol^−1^). Endogenous undecaprenyl-pyrophosphate within the membrane fraction served as the phosphate donor. The reaction mixture was incubated at 30°C for 1 h. Reaction products were analysed by TLC in a solvent system of chloroform, pyridine, 88% formic acid, water (30:70:16:10, v/v) and visualized by phosphorimaging analysis (Bio-Rad PMI).

### β-Galactosidase assay

Bacteria were grown overnight in LB medium, washed in the same medium to be cultured, and diluted to an OD_600_ of 0.05. FeSO_4_ was added to starting dilutions for inducing conditions when necessary. Cells were harvested at mid-exponential phase (OD_600_ of 0.5–0.6) and β-galactosidase activity of bacterial lysates was determined as described by Miller (1972). Assays were performed in triplicate, using independent colonies for each culture.

### MIC assay using polymyxin B

Cells were grown overnight followed by a 1:100 dilution in LB. Cells were cultured up to exponential phase, diluted to an OD_600_ of 0.05 and applied to LB agar. Quantitative MIC values were determined using ETest gradient Polymyxin strips (AB Biodisk) after 24 h.

### Mass spectrometry of lipid A species

Typically, 200 ml cultures of each strain were grown at 37°C until cultures reached an OD_600_ of ∼1.0. Lipid A was released from cells and purified as previously described (Tran *et al*., 2006). The lipid A species were analysed as previously described (Hankins and Trent, 2009) by the UT-Austin Analytical Instrumentation Facility Core using a MALDI-TOF/TOF (ABI 4700 Proteomics Analyser) mass spectrometer equipped with an Nd:YAG laser (355 nm) using a 200Hz firing rate. The spectra were acquired in negative ion linear mode and each spectrum represented the average of a minimum of 4000 shots. The matrix used was a saturated solution of 6-aza-2-thiothymine in 50% acetonitrile and 10% tribasic ammonium citrate (9:1, v/v). The samples were dissolved in chloroform-methanol (4:1, v/v) and deposited on the sample plate, followed by an equal portion of matrix solution (0.3 ml).
