# Supplementary material for: Activation of PmrA inhibits LpxT-dependent phosphorylation of lipid A promoting resistance to antimicrobial peptides
Source: Mol Microbiol. 2010 Apr 9;76(6):1444–60. doi: 10.1111/j.1365-2958.2010.07150.x (PMC2904496; doi:10.1111/j.1365-2958.2010.07150.x)
Supplement: Supplementary file 1 [file mmi0076-1444-SD1.pdf]

Supplemental material

Figure S1. LpxT-dependent lipid A modification is impaired in PmrA constitutive (*pmrA*^{c}) *E. coli*. Strains WD101 (W3110, *pmrA*^{c}), WDeptA (pmrA^c, Δ*eptA*), WDarnT (*pmrA*^{c} Δ*arnT*), WDeptAarnT (pmrA^c, Δ*eptA*, Δ*arnT*) and WDlpxT (pmrA^c, Δ*lpxT*) were grown in LB medium in the presence of $^{32}P_i$ followed by lipid A extraction. The lipid A profile in WD101 strain and its derivatives does not show LpxT-dependent lipid A modification. W3110 strain was used as control for 1-PP lipid A synthesis.

Figure S2. *yeiR* and *lpxT* genes are transcribed independently. A. Schematic of a *yeiR* and *lpxT* genomic arrangement in W3110 is shown. White boxes indicate a putative promoter region and primer annealing sites represented by arrows. Primers annealing to *yeiR* (RT-yeiRF and RT-yeiRR) are shown in blue. Primers annealing to *lpxT* (RT-lpxTF and RT-lpxTR) are shown in red. **B.** PCR amplifications using the indicated primers were analyzed by agarose gel electrophoresis. Left panel: internal regions of *yeiR* (197 bp) and *lpxT* (570 bp) independently, and (right) *yeiR* and *lpxT* as a single amplicon (1320 bp). Genomic DNA template was used as a positive control for primers and amplified product sizes. PCR reactions using cDNA as the template without reverse transcriptase was used to verify no DNA contamination.

Figure S3. LpxT-GFP fusion protein is a functional lipid A phosphotransferase. ^{32}P-lipid A was isolated from W3110 and CH01 (W3110, *lpxt-gfp$_{mut3}$)* and analyzed by TLC. Bacteria were grown in LB medium and ^{32}P-lipid A visualized by phosphorimaging.

Figure S4. LpxT-GFP fusion protein is not found in the soluble fraction. Strains W3110, CH01 (W3110, *lpxT-gfp*) and CH021 (Δ*pmrA*, *lpxT-gfp*) were grown in LB medium with 200 µM or without $FeSO_4$. Soluble proteins (5 µg) from the indicated strains were separated by SDS-PAGE followed by western blotting as described under “Experimental Procedures” in order to demonstrate that LpxT-GFP is not found within the soluble fraction of bacterial lysates. The 29.3-KDa GFPuv protein found in the soluble fraction of W3110 harboring the pGFPuv plasmid was used as the positive control for anti-GFP

polyclonal antibody (2 μg). Membranes from strain W3110 expressing chromosomal LpxT-GFP fusion was also analyzed in order to show the migration of the target protein. Arrows indicate the molecular mass (KDa) of protein standards.

Figure S5. Expression of LpxT *in trans* in WD101 (*pmrA^c*) results in loss of pEtN modification. ^{32}P-lipid A was isolated from cultures of WD101 and WDarnT containing plasmid pWSlpxT and analyzed by TLC. Bacteria were grown in LB medium and ^{32}P-lipid A visualized by phosphorimaging.

Table S1: Strains and Plasmids

Strain or Plasmid	Description*	Reference
**Strains**		
E. coli		
DY330	W3110 Δ*lacU169 gal490* l *c1857* Δ(*cro-bioA*) *endA1 gyrA96*(nalR) *thi-1 recA1 relA1 lac* glnV44	(Yu *et al.*, 2000)
XL-1 blue	F'[::Tn10 *proAB*+ $lacI^q$ Δ (*lacZ*)M15]	Stratagene
W3110	Wild type, F^- l^- rph-1 INV(*rrnD, rrnE*)1 *rph-1*	*E. coli* Genetic Stock center (Yale)
WD101	W3110 constitutive *pmrA* mutant, Pmb^R	(Trent *et al.*, 2001)
CH01	W3110 LpxT-GFP_{mut3} translational fusion, Kan^R	This study
DMEG3	BW25113 ΔlpxT::cat, Cam^R	(Touze *et al.*, 2008)
MST01	W3110, Δ*lpxT*	This study
CH020	W3110 Δ*pmrA::nptII,* Kan^R	This study
CH021	CH020 LpxT-GFP_{mut3} translational fusion, Kan^R,	This study
CH030	W3110, Δ*eptA*	This study
CH040	W3110, Δ*arnT::cat,* Cam^R	This study
CH034	CH030, Δ*arnT::cat,* Cam^R	This study
WDeptA	WD101 Δ*eptA::nptII*, Kan^R	This study
WDarnT	WD101 Δ*arnT::cat*, Cam^R	This Study
WDeptAarnT	WDeptA, Δ*arnT::cat*, Kan^R, Cam^R	This study
WDlpxT	WD101 Δ*lpxT::cat*, Cam^R	This Study
S. typhimurium		
LT2	Wild type	ATCC
CH05	LT2, Δ*lpxT::cat,* Cam^R	This study
CH06	LT2, Δ*arnT::cat,* Cam^R	This study
CH07	LT2, Δ*eptA*::*nptII*, Kan^R	This study
CH057	CH05, Δ*eptA*::*nptII*, Kan^R	This study
**Plasmids**		
pWSK29	Low copy number, Amp^R	(Wang & Kushner, 1991)
p3174	GFP_{mut3} template vector, Kan^R	(Gerlach *et al.*, 2007)
pCP20	FLP-expressing vector, Amp^R, Cam^R	(Datsenko & Wanner, 2000)
pGFPuV	Cloning vector, green fluorescent protein variant (GFP_{uv}), Amp^R	Clontech
pRS415	multicopy lac-based cloning vector, $Amp^{R,}$	(Simons *et al.*, 1987)
pKD3	*cat* gene template plasmid, Cam^R, Amp^R	(Datsenko & Wanner, 2000)
pKD4	*nptII* gene template plasmid, Kan^R, Amp^R	(Datsenko & Wanner, 2000)
pKD46	Red recombinase expression plasmid, temperature sensitive, Amp^R	(Datsenko & Wanner, 2000)
pACYC184	pA15A origin of replication, low copy number cloning vector, Tet^R, Cam^R	Novagen
pWSlpxT	*lpxT* cloned into pWSK29, Ap^R	This study

pWSlpxT$_{H190A}$	pWSlpxT encoding a mutated *lpxT*$_{(A190)}$, Ap^R	This study
pMST2	250-bp promoter *lpxT* region cloned into pRS415, Ap^R	This study
pCH02	300-bp promoter *eptA* region cloned into pRS415, Ap^R	This study
pACeptA	eptA cloned into pACYC184 Tet^{R,} Cam^R	This study

*Resistance (R) to: Amp (Ampicillin), Cam (Chloramphenicol), Kan (Kanamycin), Tet (Tetracycline), Pmb (polymyxin B)

Table S2. List of primers

Primers	Sequence (5'-3')	Purpose
P1pmrA	CTGCAAACTTGCAGGAGAGTGAGTGAATGAAAATTCTG ATTGTTGAAGACGTGTAGGCTGGAGCTGCTTC	*pmrA* deletion
P2pmrA	TCGGCGCAGAAAATGCATCAGATTCAATTAGTTTTCCT CATTCGCGACCAGCATATGAATATCCTCCTTA	*pmrA* deletion
P1arnT	GGACGTGAAGGCTGGCTGGGTTGCCAACAAATTGCGG GTAGTCGCTGATGGTAGGCTGGAGCTGCTTCG	*arnT* deletion
P2arnT	CAAGCTGGCAAAGACTAATGTTAGCCAGATCATTTGGG ACGATACTGAATATGGGAATTAGCCATGGTCC	*arnT* deletion
P1eptA	ACTTTGTTCGATGGAAACACCGTGATGTTGAAGCGCCT ACTAAAAAGACCCGTGTAGGCTGGAGCTGCTTC	*eptA* deletion
P2eptA	CGTATCGTCTTCAACAATCAGAATTTTCATTCACTCACT CTCCTGCAAGT	*eptA* deletion
LpxTgfpF	CCTTTTTTGACAAATCACTACCAGGAAAAAACAAACATT TCCAAAACAAAAGTAAAGGAGAAGAACTTTTC	*lpxT-gfp* fusion
LpxTgfpR	ATTATCCTCACTATAAAAATAACCCTGATGATGTTAATT ACTGTGAGTTACGTGTAGGCTGGAGCTGCTTC	*lpxT-gfp* fusion
LpxTA190-S	CCAGAGTAATGATTGGCGCAGCCTGGTTTACTGACAT CATTG	*lpxT* site-specific mutation
LpxTA190-A	CAATGATGTCAGTAAACCAGGCTGCGCCAATCATTACT CTGG	*lpxT* site-specific mutation
PeptAF	GTATTCCGGAATTCGGGTAAAGCACGCCCGGCATATC TGGC	*eptA* promoter cloning into pRS415
PeptAR	TCTATCGCGGATCCGCACGGTGTTTCCATCGAACAAA GTGC	*eptA* promoter cloning into pRS415
PlpxTF	GCGCGCGAATTCGGGCGCGACTTGCACCGG	*lpxT* promoter cloning into pRS415
PlpxTR	GCGCGCGGATCCATTTTCTCTAATTATCTT	*lpxT* promoter cloning into pRS415
LpxTF	ACTTATCGCGGATCCGCGTAAGGAGAGAAAATATGATT AAAAATTTGCCGC	*lpxT* cloning into pWSK29
LpxTR	ATGTAATCCGGAATTCCGGTTATTTGTTTTGGAAATGTT TGTTTTTTCC	*lpxT* cloning into pWSK29
50EptAF	GCGCGCAAGCTTTAATTTTGCTTTGCGAGC	*eptA* cloning into pACYC184
50EptAR	GCGCGCGGATCCTCATTCACTCACTCTCCT	*eptA* cloning into pACYC184
RT-lpxTF	AGTGTTGTTGAATATTGTCGGCC	cDNA-PCR of *lpxT*
RT-lpxTR	CCAATCATTACTCTGGGAAATGC	cDNA-PCR of *lpxT*
RT-yeiRF	ACCTTACTGCGTCAGGGAAA	cDNA-PCR of *yeiR*
RT-yeiRR	CAGCTGGTCACGGAAGTTTT	cDNA-PCR of y*eiR*
P1LpxTST	TAAGGTTGCCACCGTTTAAAAACTATGGCGAAATGACG ATGAAAACCCGCTGTAGGCTGGAGCTGCTTCG	*lpxT* deletion in LT2
P2LpxTST	GGTAATGTTAATTACATAGGTTATTTGTTTAAAATTTGT TTATTTCCGCCATGGGAATTAGCCATGGTCC	*lpxT* deletion in LT2
P1EptAST	GCCTGAACATTGCGTTCTACAAGCAGGTACTACAAGAC CTACCGTTAAACGTGTAGGCTGGAGCTGCTTC	*eptA* deletion in LT2
P2EptAST	TGCGTATCCGGCGCAATGGAATACGGCAAACCGTGTA AATAAACGCCATTCATATGAATATCCTCCTTAG	*eptA* deletion in LT2

P1ArnTST	GAAGGTTGGCTGGGGTGTCAACAGGCAGCGAGCGCC<br>TCATGATGAAATCGTGTAGGCTGGAGCTGCTTCG	*arnT* deletion in LT2
P2ArnTST	AGACTGGCAAGCACCAGAACGACGCCGATCATTTAGG<br>CCGATACTGAATTATGGGAATTAGCCATGGTCC	*arnT* deletion in LT2
UpmrAF	CACCAGCCTGGTTTATCTTT	Verification of Δ*pmrA*
DpmrAR	CAGGCTGACCATAAAGACGC	Verification of Δ*pmrA*
UarnTF	GATTGGTCGGGATGTGAAAG	Verification of Δ*arnT*
DarnTR	CAGGTTGCCTGTTTCTGACA	Verification of Δ*arnT*
UeptAF	GTAAAGCACGCCCGGCATAT	Verification of Δ*eptA*
DeptAR	CTTTGTCGCGCAGATTGTG	Verification of Δ*eptA*
UlpxT	CGAAGGGCTGGTGCGAATCA	Verification of *lpxT*-*gfp*$_{mut3}$ fusion
GFP-Fu	GGTGATGTTAATGGGCACAA	Verification of *lpxT*-*gfp*$_{mut3}$ fusion
UlpxTST	CAGCCGGGTTGAACTTATCT	Verification of Δ*lpxT* in LT2
DlpxTST	CAGCACGGGTCAAATTAAGC	Verification of Δ*lpxT* in LT2
UeptAST	GCGCTGTATGCGTTGAACTA	Verification of Δ*eptA* in LT2
DeptAST	CCCTAAATCCAGCACCATCA	Verification of Δ*eptA* in LT2
UarnTST	TGAAGATTTGCTCGTTCGTG	Verification of Δ*arnT* in LT2
DarnTST	AGGGTTTGTAGCACCAGCAG	Verification of Δ*arnT* in LT2

References

Datsenko, K. A. & B. L. Wanner, (2000) One-step inactivation of chromosomal genes in *Escherichia coli* K-12 using PCR products. *Proc Natl Acad Sci U S A* **97**: 6640-6645.

Gerlach, R. G., S. U. Holzer, D. Jackel & M. Hensel, (2007) Rapid engineering of bacterial reporter gene fusions by using Red recombination. *Appl Environ Microbiol* **73**: 4234-4242.

Simons, R. W., F. Houman & N. Kleckner, (1987) Improved single and multicopy lac-based cloning vectors for protein and operon fusions. *Gene* **53**: 85-96.

Touze, T., A. X. Tran, J. V. Hankins, D. Mengin-Lecreulx & M. S. Trent, (2008) Periplasmic phosphorylation of lipid A is linked to the synthesis of undecaprenyl phosphate. *Mol Microbiol* **67**: 264-277.

Trent, M. S., A. A. Ribeiro, W. T. Doerrler, S. Lin, R. J. Cotter & C. R. Raetz, (2001) Accumulation of a polyisoprene-linked amino sugar in polymyxin-resistant *Salmonella typhimurium* and *Escherichia coli*: structural characterization and transfer to lipid A in the periplasm. *J Biol Chem* **276**: 43132-43144.

Wang, R. F. & S. R. Kushner, (1991) Construction of versatile low-copy-number vectors for cloning, sequencing and gene expression in *Escherichia coli*. *Gene* **100**: 195-199.

Yu, D., H. M. Ellis, E. C. Lee, N. A. Jenkins, N. G. Copeland & D. L. Court, (2000) An efficient recombination system for chromosome engineering in *Escherichia coli*. *Proc Natl Acad Sci U S A* **97**: 5978-5983.

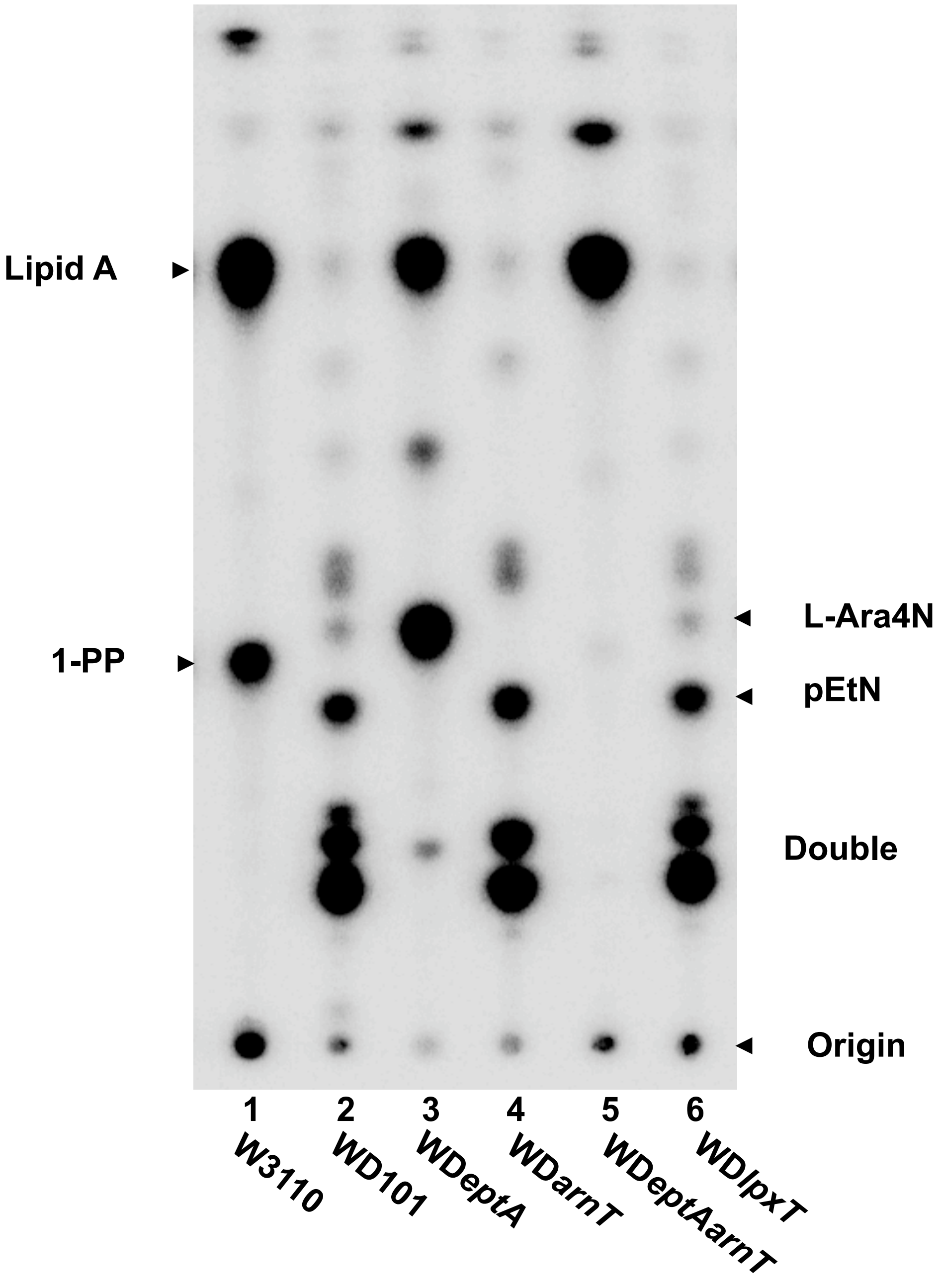
Lipid A
1-PP
L-Ara4N
pEtN
Double
Origin
1
2
3
4
5
6
W3110
WD101
WDeptA
WDarnT
WDeptAarnT
WDlpxT

Figure S1

A

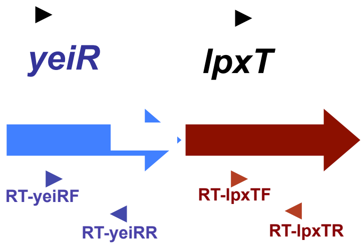

B

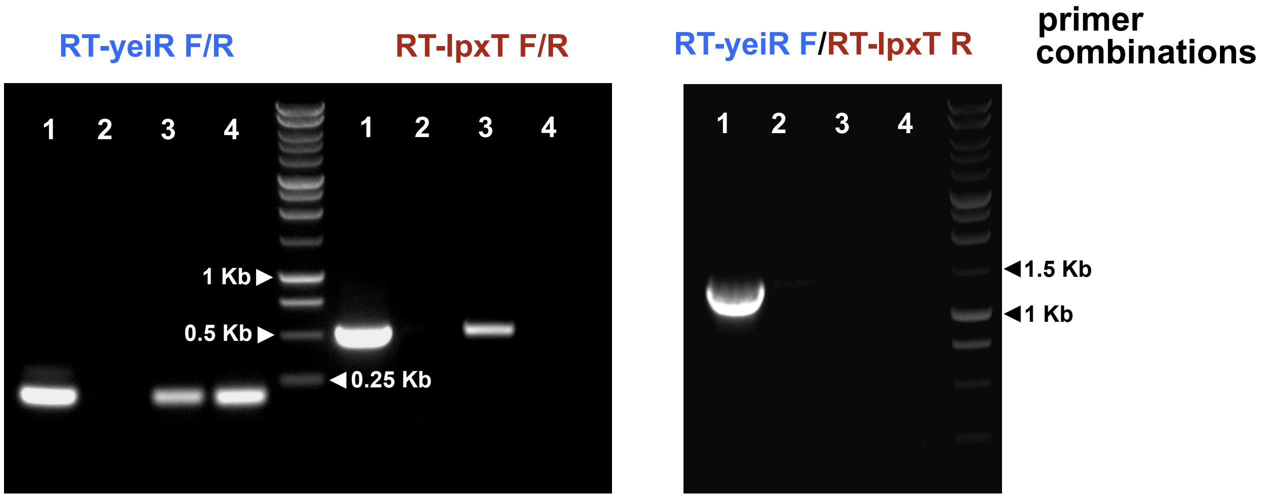

Lane	PCR template	Reverse transcriptase
1	Genomic DNA, W3110	+
2	cDNA, W3110	-
3	cDNA, W3110	+
4	cDNA, W3110 ΔlpxT	+

Figure S2

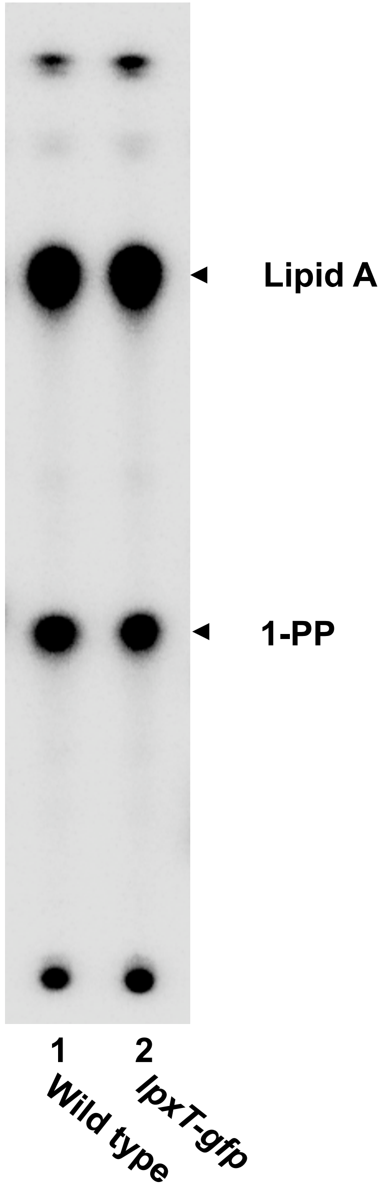

Figure S3

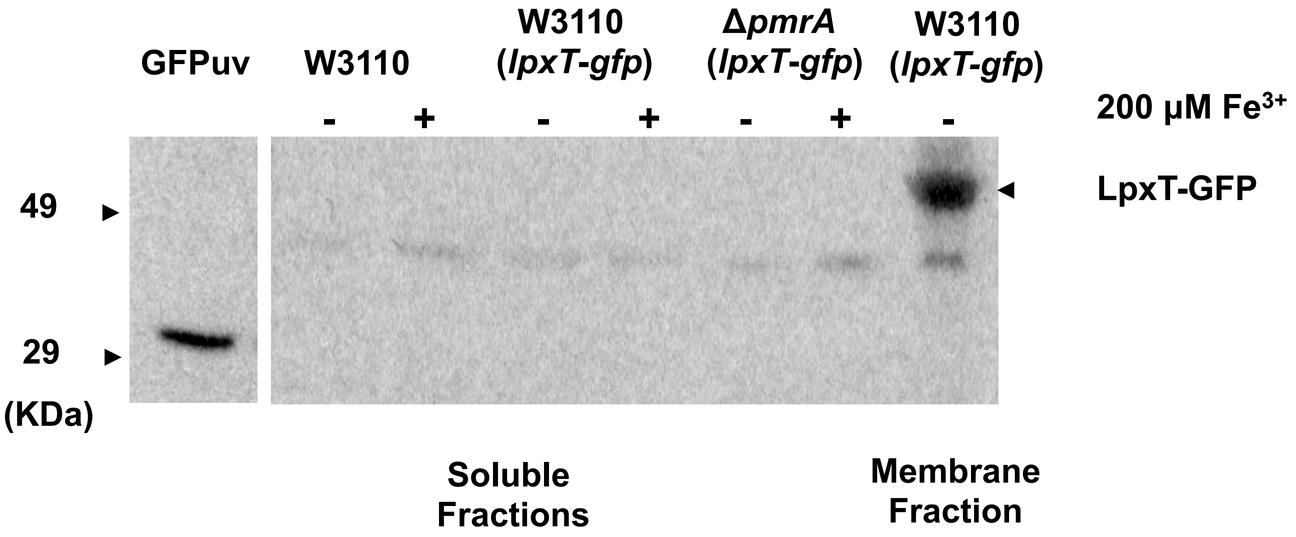
GFPuv
W3110
W3110 (lpxT-gfp)
ΔpmrA (lpxT-gfp)
W3110 (lpxT-gfp)
200 µM Fe3+
- + - + - + -
49
29
(KDa)
LpxT-GFP
Soluble Fractions
Membrane Fraction

Figure S4

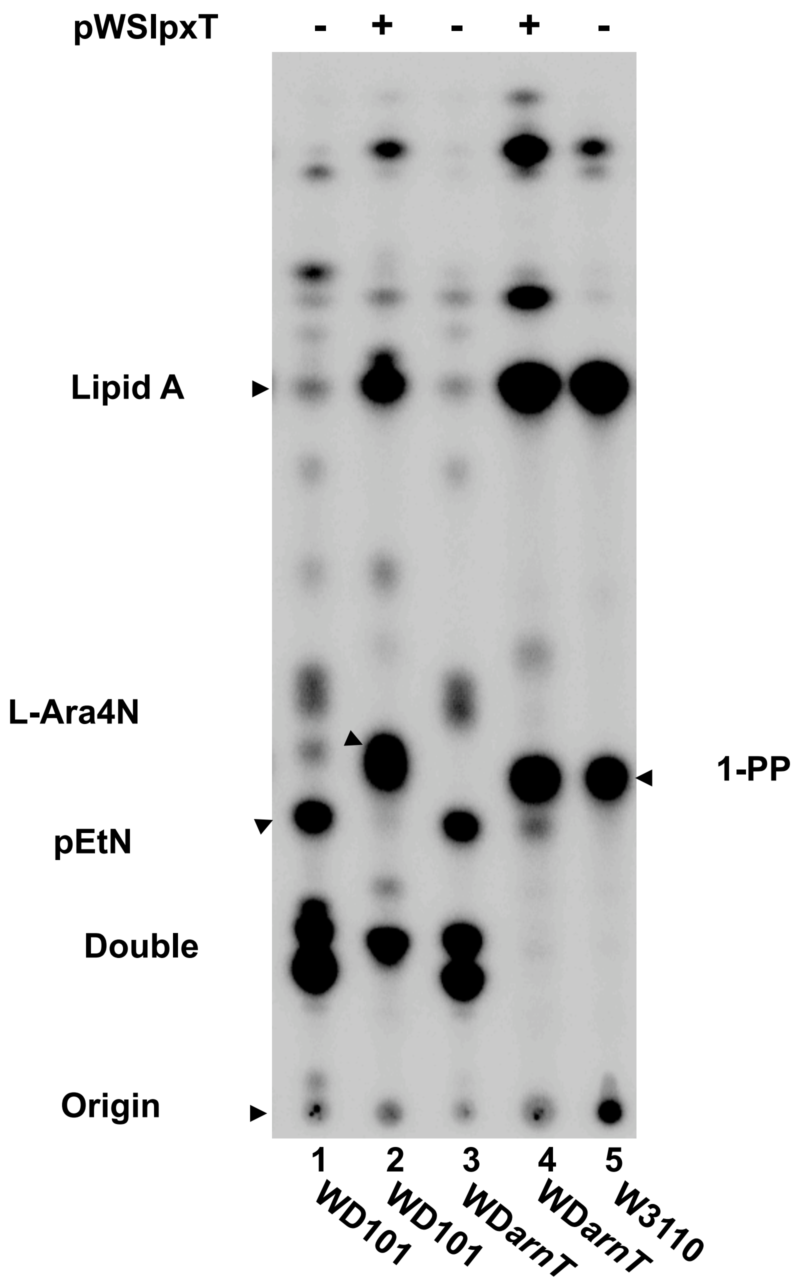
pWSlpxT
-
+
-
+
-
Lipid A
L-Ara4N
pEtN
Double
Origin
1-PP
1
2
3
4
5
WD101
WD101
WDarnT
WDarnT
W3110

Figure S5
